# A global mass budget for positively buoyant macroplastic debris in the ocean

**DOI:** 10.1038/s41598-019-49413-5

**Published:** 2019-09-12

**Authors:** Laurent Lebreton, Matthias Egger, Boyan Slat

**Affiliations:** 1 The Ocean Cleanup Foundation, Rotterdam, The Netherlands; 2 The Modelling House Limited, Raglan, New Zealand

**Keywords:** Environmental sciences, Ocean sciences

## Abstract

Predicted global figures for plastic debris accumulation in the ocean surface layer range on the order of hundreds of thousands of metric tons, representing only a few percent of estimated annual emissions into the marine environment. The current accepted explanation for this difference is that positively buoyant macroplastic objects do not persist on the ocean surface. Subject to degradation into microplastics, the major part of the mass is predicted to have settled below the surface. However, we argue that such a simple emission-degradation model cannot explain the occurrence of decades-old objects collected by oceanic expeditions. We show that debris circulation dynamics in coastal environments may be a better explanation for this difference. The results presented here suggest that there is a significant time interval, on the order of several years to decades, between terrestrial emissions and representative accumulation in offshore waters. Importantly, our results also indicate that the current generation of secondary microplastics in the global ocean is mostly a result of the degradation of objects produced in the 1990s and earlier. Finally, we propose a series of future emission scenarios until 2050, discussing the necessity to rapidly reduce emissions and actively remove waste accumulated in the environment to mitigate further microplastic contamination in the global ocean.

## Introduction

Since mass production of synthetic polymers started in the 1950s, plastic waste has been accumulating and degrading in terrestrial and oceanic environments^1^. Particularly, positively buoyant plastic objects are accumulating at the surface of the oceans, transported by currents, wind and waves, reaching remote subtropical oceanic gyres^2–5^. In 2010, annual emissions of plastic waste from land into the ocean were estimated to range between 4.8 and 12.7 million metric tons^6^. Integrated from the 1950s when plastic was first introduced in our societies, and assuming emissions proportional to global plastic production, the total accumulated mass on the ocean surface layer should be as high as tens of millions metric tons. Synthetic polymers with a density lower than sea water represent over 65.5% of the current global plastic production^7^. Yet, a major fraction of positively buoyant plastic is missing as current estimates of >250,000 metric tons^8^ are far from the predicted tens of millions of metric tons that should be floating in the global ocean by now.

The answer to the missing plastic question could be the combination of three possible explanations. Firstly, the input of plastic into the ocean could be overestimated. Evaluation of inputs rely on reported country-scale statistics on municipal waste generation^9^ for which a fraction is assumed to reach the ocean. However, the dynamics of release into the marine environment are poorly known. Other assessments considering emissions from rivers as a function of rainfall and plastic waste generation predict smaller inputs, yet still on the orders of million metric tons^10,11^. Secondly, the total plastic mass currently floating on the ocean surface could be underestimated. Commonly, surface concentrations of plastic pollution are monitored using neuston nets (generally <1 m sampling width) with mesh size typically in the sub-millimeter scale, requiring large sampling effort due to spatial and temporal heterogeneity^12^. This data is then coupled with global dispersal models to assess the total mass of plastic debris on the ocean surface layer^13,14^. Previous work has demonstrated that combining surface trawl data with visual sightings for the occurrence of larger debris (>200 cm) results in greater estimates^8^. More recently, a study using larger surface trawls (6 m sampling width) and aerial imagery (~360 m sampling width) instead of visual sightings to calculate the mass contribution of debris respectively >5 cm and >50 cm in size, has shown that accepted concentration figures could be underestimated by a factor of four to sixteen^15^ in the North Pacific. This may also be true in other parts of the world but even with a four to sixteen fold underestimation, this would result in a predicted standing mass of several hundreds of thousands to a few million metric tons, which is still one to two orders of magnitude lower than the tens of million metric tons of plastic that are expected to have entered the ocean since the 1950s. Thirdly, positively buoyant plastic could be removed from the sea surface. Floating plastic debris at sea undergoes fouling that may result in a loss of buoyancy in seawater. If the debris density increases sufficiently, buoyant plastic will be transported to deeper water depths. In deeper and different environments (e.g. aphotic zone), debris may experience rapid defouling and resurface for a repetition of the same cycle of events^16^. However, in shallower depths, debris could eventually reach the marine benthic sediment or directly strand on shorelines. Furthermore, plastic at sea is degrading into smaller particles due to photodegradation, mechanical abrasion and oxidation^17^. The observed size distribution of plastic debris collected at the ocean surface shows that the smaller fraction of microplastics (<0.1 cm) is underrepresented when compared to expected degradation rates from larger objects^8,13^. These smaller particles could potentially be disappearing faster from the sea surface from ingestion by marine life^18^ and incorporation into marine snow^19^, aggregation^20,21^, or sinking from biofouling^22^. Unfortunately, current sampling techniques are very limited for the *in-situ* detection of sub-millimeter sized particles and far less is known for particles smaller than microplastics (<0.05 cm). Assuming the distribution of plastic mass per size class had not reached equilibrium, one could also argue that the smaller size fraction could still be in formation but remains underrepresented as the emissions overwhelms degradation rates.

Researching these questions, a recent study presented a global model for emission, degradation and settling of macroplastics (>0.5 cm) and microplastics (<0.5 cm) in the ocean. Assuming no settling for macroplastics, the results suggested that 99.8% of the plastic mass that has entered the marine environment since 1950 has degraded into micro- and nanoplastics and has subsequently settled below the surface layer^23^. The authors predicted that under a zero-emission scenario, almost all plastic would be removed from the ocean surface layer within three years. Yet, accumulation of plastic in the global ocean has mostly been mapped for surface waters but very little information is known on where the underwater plastic may have accumulated. Moreover, while the latter explanation could solve the question of the missing ocean plastic, it raises new questions. Dispersal models and observations suggest that debris released from terrestrial sources and accumulating in offshore oceanic gyres requires on average a minimum of several years to reach such remote oceanic areas^24,25^. Thus, if positively buoyant plastic were persisting less than three years on the surface layer, accumulation in offshore subtropical gyres would not occur. Furthermore, a recent study analyzing the age of plastic objects found in the North Pacific subtropical gyre by identifying production dates on collected macroplastics at sea reported a significant number of decades-old objects, dating as far back as the 1970s^15^. The degradation of plastic initiated by solar UV radiation is severely retarded when floating in seawater^17^. While the production date does not necessarily inform on the disposal date, the relative age distribution of objects found at sea, assuming a sufficiently large sample size, should be representative of the discard of plastic objects from the different consumer and market sectors, and their associated product lifetime^7^.

Here, we propose a new global ocean surface mass balance budget model for positively buoyant macroplastics. The principal objective of this study is to identify the key processes governing the fate of marine litter based on field evidence and to orient future research. Different model parameterizations are tested to predict the mass of positively buoyant plastic in offshore surface waters. We constrain our model parameters with field data, dispersal model outputs and recent estimates from the literature. We create a simple whole-ocean emission-transport-degradation model, by including probabilities of debris stranding/settling and recirculation into coastal environments. We propose a convergent model to explain (1) the discrepancies between current accepted figures for plastic emissions and standing stock on the ocean surface layer, and (2) the occurrence of decades-old debris in subtropical oceanic gyres. Using our convergent parameterized model, we predict future mass of positively buoyant plastic in the ocean under several emission scenarios.

## Methods

### Global ocean surface mass balance model

We introduce a simple box model to quantify buoyant macroplastics (>0.5 cm) at the surface of the global ocean. We consider synthetic polymer production data from 1950 to 2015 and compute the fraction of positively buoyant polymers (65.5% of total) used by different market sectors associated with product lifetime distributions^7^. Starting in 1950 and for every year, we compute the plastic population age distribution of material mass that is reaching end of lifetime and is therefore discarded. We define the global mass of plastic D, produced in year *y*_0_ and discarded in year *y* as follow:

1 $$D(y,{y}_{0})=\mathop{\sum }\limits_{\sigma }^{\begin{array}{c}market\,\\ sectors\end{array}}\,Production({y}_{0})\,\ast \,Market\,Share(\sigma )\,\ast \,LifeTime(y-{y}_{0},\sigma )$$

With *Production*(*y*_0_), global plastic production for year *y*_0_, *MarketShare*(*σ*), percentage of market share in global plastic production for sector *σ* and, *Lifetime*(*y-y*_0_, *σ*), probability density function of product lifetime for sector *σ* and plastic age *y-y*_0_ as proposed by Geyer *et al*.^7^ with market sectors including “Packaging”, “Transportation”, “Building and Construction”, “Electrical/Electronic”, “Consumer & Institutional Products”, “Industrial Machinery” and “Other”. More information on the market share and probability density functions for lifetime of plastic objects is provided in Supplementary Table 1.

Some of the discarded plastic waste may enter the global ocean in coastal areas^6^. Once at sea, buoyant plastic may strand back on the shoreline or sink from fouling-induced loss of buoyancy. At deeper depths, debris may experience rapid defouling followed by resurfacing as floating debris^16^. In shallower depth, however, debris has a higher chance to reach the seabed. In this framework, we define the coastal waters as the area with bathymetry between high tide line and the euphotic depth (typically shallower than 200 m water depth). Our model considers that when in the coastal environment a fraction of floating plastic mass is captured by the landmass, undergoing repeated episodes of stranding and release at the shoreline or settling and resurfacing from the seabed. Some of the floating plastic remaining at the surface may be transported to offshore waters. As time passes, the mass of stranded, settled and floating plastic degrades into microplastics, thus leaving the model domain. Our model primarily focuses on buoyant macroplastics and considers mass loss from degradation into microplastics as a permanent sink.

**Figure 1:**
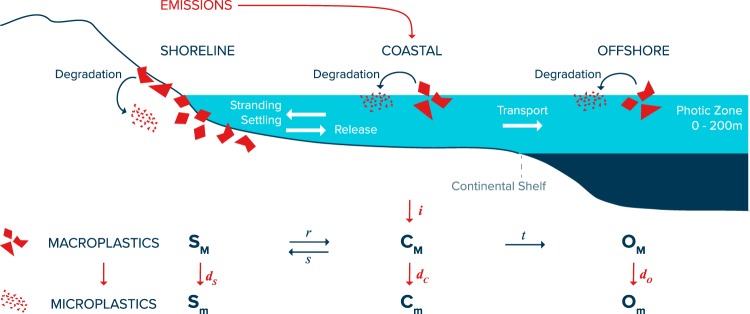
Predicting quantities of positively buoyant macroplastics (>0.5 cm) in the ocean environment using a global ocean emission-transport-degradation model. Every year, a fraction *i* of discarded plastic material is emitted into the coastal surface layer (C_M_). Material present in coastal waters can strand or settle around shorelines (S_M_) with probability *s* and material from S_M_ can leak back into C_M_ with release probability *r*. Material from C_M_ can escape the continental shelf and enter the ocean surface layer O_M_ with transport probability *t*. Finally, fractions *d*_*S*_, *d*_*C*_ *and d*_*O*_ of macroplastics present in the marine environment enter a permanent sink by degradation into microplastics (<0.5 cm) from the shoreline (S_m_), the coastal surface layer (C_m_) and the offshore surface layer (O_m_). The processes are repeated annually from 1950 to 2015.

We divide the global marine environment in three surface domains (Fig. 1): the shoreline (S), the coastal surface waters (C) and the offshore surface waters (O). Our model includes 6 mass compartments: S_M_, C_M_, O_M_ for macroplastics and S_m_, C_m_, O_m_ for secondary microplastics. The model conserves mass. For any year between 1950 to present, the accumulated mass of plastic that has been introduced in the global ocean is equal to the sum of these 6 mass compartments.

The model is initiated with input into coastal environments starting in 1950. Then from year *y*-1 to year *y*, we compute the net mass input of plastic produced in year *y*_0_, into the surface waters of the global coastal environment:

2 $$\Delta (y,{y}_{0})=i\,\ast \,D(y,{y}_{0})+\,r\,\ast \,(1-{d}_{S})\,\ast \,{S}_{M}(y-1,\,{y}_{0})+(1-{d}_{C})\,\ast \,{C}_{M}(y-1,{y}_{0})$$

The first term constitutes the direct inputs from discarded plastic leaking into the environment. Model parameter *i* is the annual mass fraction of discarded plastic reaching the coastal ocean. The two other terms represent respectively material released from the shoreline S_M_ and existing floating material in C_M_ left from previous years that has not yet degraded into microplastics. Model parameters *d*_*C*_ and *d*_*S*_ are regarded as the annual mass fraction of respectively floating and stranded or settled macroplastics degrading into microplastics, while *r* is the annual mass fraction of stranded or settled macroplastics that is released back into surface waters of coastal environments. The resulting mass of plastic produced in year *y*_0_ that is present in coastal surface waters during year *y* is then computed as:

3 $${C}_{M}(y,{y}_{0})=(1-s)\,\ast \,(1-t)\,\ast \,\Delta (y,{y}_{0})$$

Where *s* is the annual mass fraction of floating plastic that strands and settles around shorelines and *t* the annual mass fraction of remaining floating plastic that is transported offshore. The total mass of plastic produced in year *y*_0_ and stranded or settled around the global shoreline during year *y* is the sum of newly stranded or settled material and previously accumulated plastic that was not degraded into microplastics and not released back into coastal waters:

4 $${S}_{M}(y,{y}_{0})=(1-r)\,\ast \,(1-{d}_{S})\,\ast \,{S}_{M}(y-1,{y}_{0})+s\,\ast \,\,\varDelta (y,{y}_{0})$$

Finally, the total mass of plastic produced in year *y*_0_ and floating in offshore waters during year *y* is the sum of previously accumulated plastic that has not degraded into microplastics and new debris leaked from the coastal waters.

5 $${O}_{M}(y,{y}_{0})=\,\,(1-{d}_{O})\,\ast \,{O}_{M}(y-1,{y}_{0})+t\,\ast \,(1-s)\ast \,\varDelta (y,{y}_{0})$$

With *d*_*O*_ the degradation rate for macroplastics in offshore surface waters. For each year, the three mass sink terms are populated with input from degradation into microplastics from coastal, shoreline and offshore environments.

6 $${C}_{m}(y,{y}_{0})=(1+{d}_{C})\,\ast \,{C}_{M}(y-1,{y}_{0})$$

7 $${S}_{m}(y,{y}_{0})=(1+{d}_{S})\,\ast \,{S}_{M}(y-1,{y}_{0})$$

8 $${O}_{m}(y,{y}_{0})=(1+{d}_{O})\,\ast \,{O}_{M}(y-1,{y}_{0})$$

In this study, we assumed the degradation rates *d*_*C*_, *d*_*S*_ and *d*_*O*_ to be equal. The degradation term is called thereafter *d*.

9 $$d={d}_{C}={d}_{S}={d}_{O}$$

We note that in nature, these values may be different particularly for the shoreline where the degradation rate could be greater than in surface waters. These values will also likely differ between polymer composition and dimension of objects. We acknowledge that global degradation into secondary microplastics is far more complex than described by our model. With current available data, however, we are limited to propose a whole-ocean average degradation rate for the total macroplastic mass. Specifications of degradation rates by environments and polymer types will require more experimental research.

Another major assumption here is that model parameters do not show any interannual variability and that the dynamics of degradation, stranding, release and recirculation into the coastal environment is independent from the age and characteristics of plastic objects. A crucial parameter is the fraction *i* of new plastic waste generated on land that reaches the ocean. This parameter has a substantial influence when constraining the values of *s* (stranding on shoreline), *r* (release from shoreline) and *t* (offshore transport). We constrain parameter *i* by using estimates of global input from land into the ocean for 2010 with 4.8 to 12.7 million metric tons of input^6^. For a global plastic waste generation of 274 million metric tons in 2010^7^, this translates to a fraction of annual discarded plastic reaching the ocean ranging from *i* = 1.7–4.6%. We therefore used this reported range to define the confidence interval for the results presented here.

### Age distribution of ocean plastic

**Figure 2:**
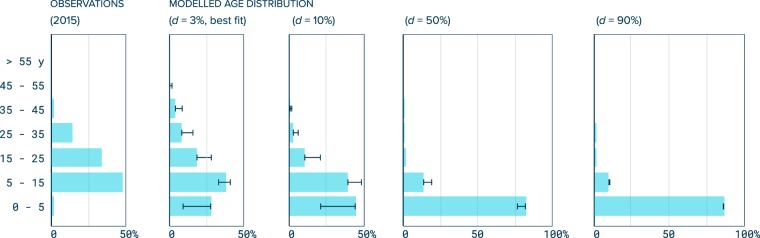
Comparison between observed and predicted plastic age distribution. Observations correspond to the relative age distribution of macroplastics collected from the North Pacific subtropical gyre in 2015^15^. This distribution is derived from production date labels identified on debris (*N* = 50). Model predicted age distributions are given for a range of the degradation variable *d* (*3%*, *10%*, *50% and 90%*) and parameters *s*, *r* and *t* set to 0%, reproducing a simple global emission-degradation model. Whiskers extend to all possible values by computing all possible combinations of *s*, *r*, and *t* varying from 0% to 100%, showing that the plastic age distribution is mostly sensitive to the degradation rate parameter *d*. We compute the least square sum for decadal distribution of observed and modelled plastic from 1950s to 2000s; minimum value is found for *d* = 3% (Supplementary Fig. 1). Pearson p-test values for d = 3%, 10%, 50%, 90% are respectively p = 0.009, 0.0133, 0.0361 and 0.0310. Underrepresentation of objects produced between 2010 and 2015 (the year when the samples were collected) is explained by the minimum time required for plastic debris to travel to oceanic gyres (~5 years).

To study the persistency of macroplastics, the age distribution of plastic in the different compartments of our model is compared to the age distribution of plastic debris collected in a large oceanic gyre. In 2015, a multivessel expedition collected marine plastics debris floating in the Great Pacific Garbage Patch located in the North Pacific subtropical gyre^15^. The expedition landed 664 kg of positively buoyant macroplastics (debris larger than 0.5 cm) back to shore. Of the 83,144 collected pieces (>0.5 cm), 427 had a recognizable inscription for which 11 languages and 50 dates of production could be identified. Here, we consider the distribution of production dates found on these samples to be representative of plastic age distribution in oceanic gyres. By exploring all possible combinations of our five model parameters ranging from 0% to 100% at every 1%, we observe that only the degradation rate *d* significantly impacts the relative age distribution of positively buoyant macroplastics in offshore surface waters (Fig. 2). This is because degradation into microplastics is the only permanent sink considered by our model i.e. it is the only natural mechanism that removes material from our model domain. Therefore, we use the observed plastic age distribution to constrain the parameter *d*. Note that when fixing parameters *s*, *r* and *t* to 0%, we reproduce a global ocean emission-degradation model for macroplastics introduced in a previous study^23^. We compare the modelled decadal distribution with our samples for a degradation rate *d* ranging from 0% to 100%. Only one plastic object with production date in the 2010s was identified in the samples which were collected in 2015. We explain this by the minimum time taken for objects to reach the area that we estimate from dispersal model trajectories to be between 5 to 10 years to be representative of sources. Therefore, accounting for a minimum delay of 5 years, we compared observed and modelled decadal distributions of production dates from the 1950s to the 2000s. We computed the sum of squared residuals between observed and modelled age distribution by decades and varied model parameter d for minimization. Best fit was found for *d* = 3% of mass of positively buoyant macroplastics annually degraded into microplastics (Supplementary Fig. 1). A small degradation rate is in good agreement with field experiments, estimating a mass loss ranging from 0.65% to 1.9% of total mass, depending on polymers, for samples immersed at sea for a period of 12 months^26^.

### Model sensitivity analysis

**Figure 3:**
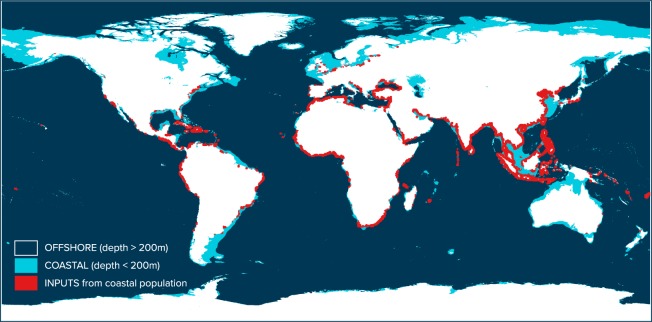
Lagrangian dispersal model source locations and global ocean surface model domains. Amplitude and location of model particle sources are derived from predicted inputs of plastic from land into the ocean^6^ and population changes from 1993 to 2012^27^. The separation between coastal and offshore surface waters in our model is shown with areas of respectively light and dark blue color. Coastal surface waters represent the continental shelf with bottom depths shallower than the photic zone (i.e. depths <200 m).

We study model convergence by varying parameters *s*, *r* and *t* from 0% to 100%. The model is considered convergent when the confidence interval of model predicted mass floating on the global ocean surface (e.g. C_M_ + O_M_) includes values on the order of hundreds of thousand metric tons of material (i.e. <10^6^ metric tons). The model is generally converging for large values of stranding probability (*s*) and low values of offshore transport (*t*). Converging values for coastal release (*r*) are inversely proportional to the value of stranding probability (*s*). This was to be expected, given that these parameters are intrinsically connected as their difference measures the capture efficiency of the continental mass. Note that parameter *s* must be higher than *r* to reproduce accumulation on the world’s beaches. To constrain the model parameters *s* and *t*, we investigate trajectories of Lagrangian particles from a global dispersal model reproducing 20-year of surface circulation^25,15^. Particles are released from significant point sources (Fig. 3) near the coast based on population^27^ and waste management data^6^. A proportional number of particles is attributed to each country based on the estimated amount of mismanaged waste the country’s coastal population generates over a year. The particle release locations are derived from coastal population density and the timing of release is randomly distributed throughout the year. Particles are advected using different model forcing components, including sea surface currents, stokes drift and variable influences of wind.

**Figure 4:**
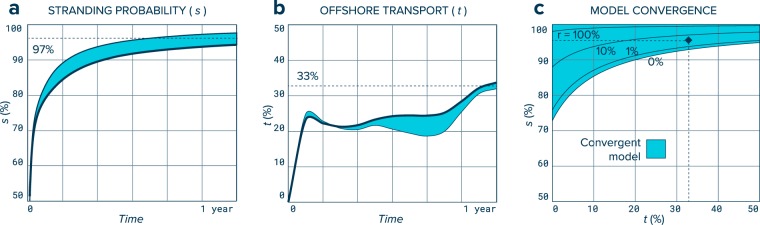
Sensitivity analysis and model convergence. (**a**) Stranding probability (*s*) determined from Lagrangian particle trajectories starting in coastal environments. The value of *s* is defined as the percentage of particles present in the model that have spent two consecutive days in close proximity (<1/16°) to the shoreline. Intervals correspond to different forcing scenarios with influence of wind ranging from 0% (thick dark line) to 2% (thin dark line) of 10 m height wind speed. Probability of stranding increases with windage coefficient. (**b)** Offshore transport (*t*) estimated from the same Lagrangian trajectories. The parameter *t* is defined as the percentage of model particles that are located outside the continental shelf (>200 m water depth) after one year of release. Intervals also correspond to different wind forcing scenarios ranging from 0% (thick dark line) to 2% (thin dark line). Generally, the probability of transport to offshore waters decreases with windage coefficient. (**c)** Sensitivity analysis and model convergence. Parameters *s*, *t*, and *r* vary from 0% *t*o 100%. The model is considered convergent (blue colored area) when the confidence range for mass estimate on the global ocean surface layer (O_M_ + C_M_) overlaps with values below 10^6^ metric tons, assuming a degradation rate *d* = 3% and emission rates *i* = 1.7–4.6%. Our model is converging with estimated value *s* = 97%, *t* = 33%, and *r* = 1% (black diamond).

For stranding probability (*s*), we follow particles from their day of release until they spend two consecutive days near the shoreline. The model parameter (*s*) is defined as the fraction of model particles that have spent at least two consecutive days near the shoreline after one year since their initial release over the total number of particles present in the model. A particle is considered near the shoreline when it is located at a distance smaller than the hydrodynamic model cell size from a land cell (1/16°, several kms depending on latitude). With no wind influence and after one year, 96% of the 2,510,918 particles investigated have spent at least two consecutive days near the shoreline. This value increases when adding wind forcing, for a windage coefficient of 2% (of 10 m height wind speed value), 98% of particles have transited around the coast for at least two days. This increase in beaching probability for high windage debris is in good agreement with observations reporting mainly low-windage debris accumulating in oceanic gyres^24^. Here, we considered that if a particle spends more than two consecutive days in contact to the shoreline it is likely stranded as it would have gone through at least one full tidal cycle. We used these results to define the stranding probability in our model with *s* = 96–98%. For offshore transport probability (*t*), we locate the fraction of particles that stay on the continental shelf one year after release. The continental shelf is defined in our model by a water depth shallower than 200 m. We used gridded bathymetry data from the General Bathymetric Chart of the Ocean^28^ to determine ocean water depth. After one year of release, between 32% and 34% of modelled non-beaching particles have escaped the continental shelf. Values decrease with windage coefficient, except for the first month which we attribute to a rapid presorting of model particles depending on emissions location. We used these results to estimate the annual offshore transport probability with *t* = 32–34%. Using midpoint values for *s* and *t* (i.e. 97% and 33%, respectively), a coastal release parameter *r* = 1% results in convergence (Fig. 4) and explains the discrepancies between emissions estimate and observed mass on the global ocean surface layer. An overview of the model parameters, with description and selected values is given in Table 1.

**Table 1:** Whole-ocean model parameters description and corresponding selected values for this study.

Variable	Description	Value in this study	Source
*i*	Annual mass fraction of plastic discarded on land that reaches the ocean	1.7–4.6%	Fitted from^6,7^
*s*	Annual mass fraction of plastic in coastal environment that strands or settles around the shoreline.	97%	This study, dispersal model outputs.
*r*	Annual mass fraction of stranded or settled plastic that resurfaces to coastal waters.	1%	Fitted from^8^
*t*	Annual mass fraction of plastic in the coastal environment that is transported offshore.	33%	This study, dispersal model outputs.
*d* (*d*_*S*_, *d*_*C*_, *d*_*O*_)	Annual mass fraction of plastic that leaves the model domain by degrading into smaller microplastics.	3%	Fitted from^15^

## Results

### Mass and age of buoyant plastics in the ocean environment

Under this convergent parameterized model, we provide an alternative explanation for the large differences between total predicted emissions of buoyant plastic since 1950 (70.0–189.3 million metric tons considered by our model) and total mass floating on the global ocean in 2015 (less than 1% of global emissions since 1950 with a predicted 0.61–1.65 million metric tons). A large part (66.8%) of all the buoyant macroplastic (>0.5 cm) released into the marine environment since the 1950s is stored by the world’s shoreline with debris stranded, settled and/or buried, undergoing episodes of capturing and resurfacing. We estimated for 2015, this represented 46.7–126.4 million metric tons of macroplastic. Finally, a significant mass fraction (32.3%) may already have degraded into microplastics (<0.5 cm) with 22.3–60.4 million metric tons from the shoreline and 0.29–0.80 million metric tons from the ocean.

**Figure 5:**
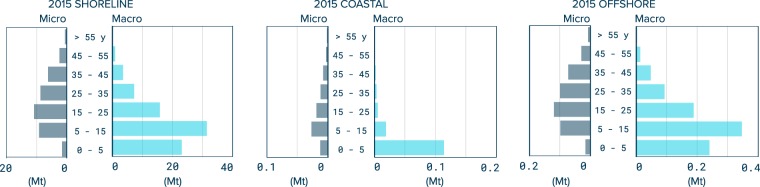
Modelled age distribution of plastic in the global ocean environment for the year 2015. Annual midpoint mass estimate by age of buoyant plastic distributed between microplastic and macroplastics at the shoreline, the coastal waters and offshore waters. Note the different scales of the x-axes.

Figure 5 shows the modeled age distribution in 2015 for plastics introduced in the ocean environment. Most buoyant plastic (79%) present in the coastal surface layer is originating from objects less than 5 years old. For the offshore surface layer, where older macroplastic objects have had more time to accumulate, plastic younger than 5 years accounts for only 26% of the buoyant plastic mass. Macroplastics older than 15 years contribute nearly half of the total mass (47%). Finally, the modeled age distribution of secondary microplastics generated from the degradation of macroplastics shows that most (74%) of the degraded plastic mass in the ocean comes from objects produced in the 1990s (27%) and earlier (47%).

### Future emission scenarios

**Figure 6:**
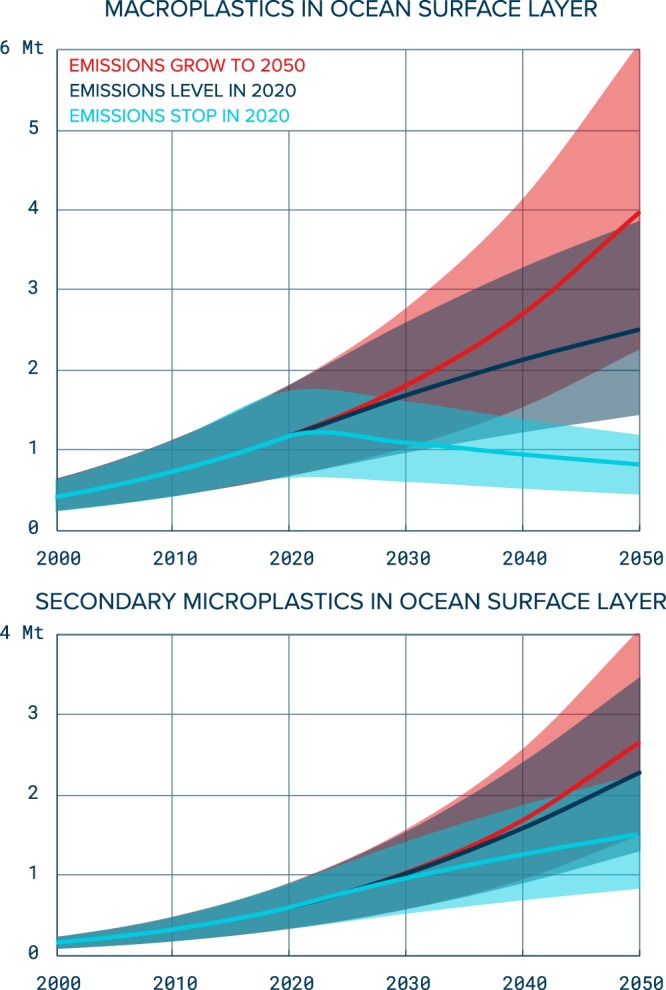
Future projections for accumulated mass of buoyant macroplastics (top) and degraded material (microplastics, bottom) from the ocean surface layer under three scenarios for emissions. (Red) Emissions are increasing at average 2005–2015 growth rate, (Dark blue) emissions are constant from 2020 and (Light blue) emissions are stopped from 2020. Solid lines represent mid-point estimates while shaded areas represent uncertainties (see Section 2.1).

Using our convergent parameterized model, we assessed future scenarios from 2015 to 2050. We defined three source scenarios with (1) emissions increasing with average 2005–2015 global plastic production’s annual growth rate, (2) emissions stagnating at 2020 levels and (3) no more emissions from 2020 onwards. Figure 6 shows the total projected mass per scenario and per model compartment. Our model predicts that for a business-as-usual scenario, where no effort is given to mitigating emissions, the quantities of buoyant macroplastics at the surface of the ocean and coastline could quadruple by the year 2050 (midpoint value of 4.5 million metric tons for O_M_ + C_M_ and 342.5 million metric tons for S_M_). By then, a predicted midpoint of respectively 3.0 (O_M_ + C_M_) and 231.6 (S_M_) million metric tons of plastic will have degraded into microplastics. If emissions of plastics into the oceans are kept constant from the year 2020 onwards, the mass of buoyant macroplastics on the global ocean surface and coastlines continues to increase, although at a slower rate due to the degradation of older objects into smaller particles. The latter, however, cannot compensate for annual inputs and the resulting macroplastic mass is increasing. If sources are stopped from the year 2020 onwards, floating and stranded mass of macroplastics decrease by 2050 to respectively 59% and 57% of their 2020 levels. The mass of microplastics in the ocean and on beaches, however, more than doubles from 2020 levels as material left in the environment is slowly degrading.

## Discussion

In this study, we introduce a simple global ocean surface box model for positively buoyant macroplastic that gives a plausible explanation for (1) the differences between estimated annual emissions of plastic into the marine environment and the predicted standing mass of plastic at the surface of the ocean, and for (2) the observation of significant number of decades-old objects in offshore subtropical waters. Based on field evidence and using a simple model, we offer an alternative explanation to the missing plastic question by identifying the key processes governing the fate of floating macroplastics. We argue that plastic accumulated in offshore surface waters is highly persistent. Accumulated quantities are less than initially expected because of the capacity for the global landmass to trap and filter marine litter inducing a delay -likely on the order of decades- for fragmented buoyant plastic to reach offshore accumulation zones. A rapid degradation sink term of >90% per year, as previously proposed to answer the difference between emissions and surface measurements^23^, cannot reproduce observations of plastic age distribution at sea. Instead, our model suggests that stranding, settling and resurfacing in coastal environments must be playing a major role in the removal of buoyant macroplastics from the surface of the ocean. Our model predicts that most of the plastic mass that has entered the marine environment since the 1950s has not disappeared from the ocean surface by degradation but is stranded or settled on its way to offshore waters, possibly slowly circulating between coastal environments with repeated episodes of beaching, fouling, defouling and resurfacing. Most of the modeled macroplastic mass floating in coastal waters is composed of relatively new objects while older objects are better represented in the open ocean. This is in good agreement with observations as the most common type of plastic litter found in the subtropical offshore waters are unidentified, thick, polyethylene or polypropylene plastic fragments^15^. This suggests that only certain types of plastic have the capacity to persist for a sufficient amount of time to eventually reach these accumulation zones. We hypothesize that a natural sorting for plastic debris is occurring in coastal environments, characterized with the capacity for the shoreline to capture the major part of floating material and where only a small fraction eventually escapes and accumulates in offshore waters. There is very little information on the amplitude of the different mechanisms governing the capture and the release of marine litter by the landmass. To obtain a convergent model, the stranding parameter (*s*) must be much greater than the release parameter (*r*). This informs us that the capture mechanisms should be dominating the release mechanisms, and therefore that the landmass likely is storing a major fraction of the missing plastic debris. Particularly, debris buried under sediments could be stored for unknown duration^29^ and eventually be transported to deeper water depths through sedimentary gravity flows^30^.

Furthermore, our model predicts that the microplastic contamination resulting from the formation of secondary particles in the marine environment is mostly representative of the degradation of objects from the 1990s and earlier. The comparison between our predicted value for the year 2014 (0.28–0.75 million metric tons), which does not account for direct input of microplastics from terrestrial sources, with estimates of microplastic concentration at the surface of the global ocean for the same year (0.093–0.236 million metric tons^14^), suggests that at least two-third of these microplastics have disappeared from the ocean surface layer likely by settling, ingestion, aggregation, stranding or degradation into even smaller particles. The relative removal contribution of these different mechanisms is largely unknown and would require more field observations and laboratory experiments. Thus, this current model cannot be directly translated to microplastics. Additionally, sources from terrestrial emissions of primary and secondary microplastics would need to be accounted for.

However, the framework presented here can be refined to specific polymers or market sectors as well as geographic locations to examine plastic fluxes and design efficient mitigation strategies for source reduction and cleanup. Our model was intentionally built using simple assumptions with only five varying parameters. We acknowledge that there are some uncertainties associated with using such a simple model approach as the values of these parameters likely differs between polymer or object types, or geographically and temporally. For instance, we assume the annual input rates to be directly proportional to global plastic consumption which may not be entirely true as waste generation rates may have changed since the 1950s. However, the objective of this study is to describe exchange processes by predicting orders of magnitude of mass quantities within the marine compartments considered by our model. The values of our five model parameters should be regarded as annual averages for the whole mass of positively buoyant macroplastic available in the marine environment. Here, we show how dispersal models can be used to constrain such model parameters. Assessing the age distribution of ocean plastic debris is useful when formulating mass balance budgets. We recommend the systematic collection of origin and age indicators on plastic debris found in the environment as it helps to better understand the source, transport and fate of plastic pollution at sea. Monitoring the distribution of microplastics within the water column and on the seabed may help in assessing long-term fate of microplastics at sea, thus allowing extending this current mass budget model to microplastics.

Finally, we show that comparing current emission estimates of plastic into the marine environment with data recently collected offshore is misleading, as there is a time lag of likely several years to decades between the two metrics. These results are somewhat alarming as even with an extremely ambitious scenario (no further emissions in the ocean by 2020), the level of microplastics in the ocean could double by mid-century as already accumulated plastic waste slowly degrades into smaller pieces. This information is important as it shows that mitigating microplastic pollution in the global ocean requires two major components: (1) drastically reducing emissions of plastic pollution in the coming years and (2) actively engaging in removal operations of plastic waste from the marine environment to reduce further generation of secondary microplastics for the decades to come. This conclusion can likely by applied to other natural environments. Without proper handling and management of accumulated plastic waste, the legacy of the last 70 years of throw-away society will live on through the generation of ever smaller synthetic polymer fragments in soils, freshwater ecosystems and eventually the ocean. The systematic removal of plastic waste from the natural environment should be encouraged and coordinated at a global scale.

## Supplementary information

Supplementary Material
