# Author Correction: A global mass budget for positively buoyant macroplastic debris in the ocean

**DOI:** 10.1038/s41598-020-58755-4

**Published:** 2020-01-30

**Authors:** Laurent Lebreton, Matthias Egger, Boyan Slat

**Affiliations:** 1 The Ocean Cleanup Foundation, Rotterdam, The Netherlands; 2 The Modelling House Limited, Raglan, New Zealand

Correction to: *Scientific Reports* 10.1038/s41598-019-49413-5, published online 12 September 2019

This Article contains errors.

The Competing Interests section in this Article is incorrect and should read:

“The authors are employed by The Ocean Cleanup, a non-for-profit organization that develops technology to rid the oceans of plastic.”

In addition, the text for several equations is incorrect; Equation 6,

“$${{\rm{C}}}_{{\rm{m}}}({\rm{y}},{{\rm{y}}}_{0})=(1+{{\rm{d}}}_{{\rm{C}}})\ast {{\rm{C}}}_{{\rm{M}}}({\rm{y}}-1,{{\rm{y}}}_{0})$$”

should read:

“$${{\rm{C}}}_{{\rm{m}}}({\rm{y}},{{\rm{y}}}_{0})={{\rm{C}}}_{{\rm{m}}}({\rm{y}}-1,{{\rm{y}}}_{0})+{{\rm{d}}}_{{\rm{C}}}\ast {{\rm{C}}}_{{\rm{M}}}({\rm{y}}-1,{{\rm{y}}}_{0})$$”

The text for Equation 7,

“$${{\rm{S}}}_{{\rm{m}}}({\rm{y}},{{\rm{y}}}_{0})=(1+{{\rm{d}}}_{{\rm{S}}})\ast {{\rm{S}}}_{{\rm{M}}}({\rm{y}}-1,{{\rm{y}}}_{0})$$”

should read:

“$${{\rm{S}}}_{{\rm{m}}}({\rm{y}},{{\rm{y}}}_{0})={{\rm{S}}}_{{\rm{m}}}({\rm{y}}-1,{{\rm{y}}}_{0})+{{\rm{d}}}_{{\rm{S}}}\ast {{\rm{S}}}_{{\rm{M}}}({\rm{y}}-1,{{\rm{y}}}_{0})$$”

The text for Equation 8,

“$${{\rm{O}}}_{{\rm{m}}}({\rm{y}},{{\rm{y}}}_{0})=(1+{{\rm{d}}}_{{\rm{O}}})\ast {{\rm{O}}}_{{\rm{M}}}({\rm{y}}-1,{{\rm{y}}}_{0})$$”

should read:

“$${{\rm{O}}}_{{\rm{m}}}({\rm{y}},{{\rm{y}}}_{0})={{\rm{O}}}_{{\rm{m}}}({\rm{y}}-1,{{\rm{y}}}_{0})+{{\rm{d}}}_{{\rm{O}}}\ast {{\rm{O}}}_{{\rm{M}}}({\rm{y}}-1,{{\rm{y}}}_{0})$$”

