# Supplementary material for: A global mass budget for positively buoyant macroplastic debris in the ocean
Source: Sci Rep. 2019 Sep 12;9:12922. doi: 10.1038/s41598-019-49413-5 (PMC6742645; doi:10.1038/s41598-019-49413-5)
Supplement: Supplementary file 1 — Supplementary Material [file 41598_2019_49413_MOESM1_ESM.docx]

**Supplementary Material for “A global mass budget for positively buoyant macroplastic debris in the ocean”**

Laurent Lebreton^1,2, *^, Matthias Egger^1^, Boyan Slat^1^

^1^ The Ocean Cleanup Foundation, Rotterdam, The Netherlands

^2^ The Modelling House Limited, Raglan, New Zealand

* laurent.lebreton@theoceancleanup.com


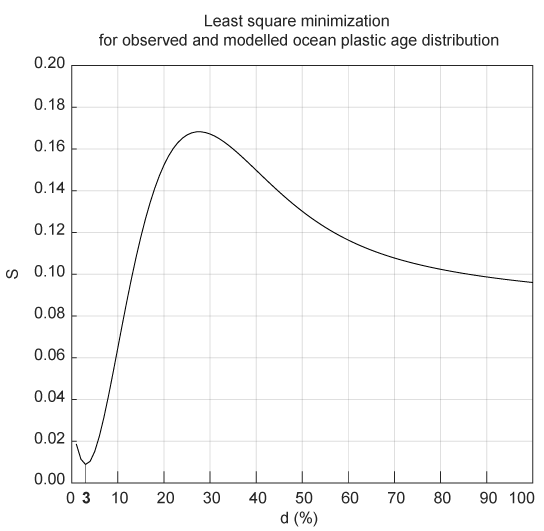


**Supplementary Figure 1: Least square minimization for observed and modelled ocean plastic age distribution.** Value of least square sum (S) for decadal distribution of observed and modelled plastic age distribution by decades from the 1950s to 2000s with degradation rate parameter *d* varying from 0% to 100%. A minimum value is found for *d* = 3%. S is computed as $S= \sum_{i}^{n} {(x_{obs}\left( i \right)-x_{mod}(i))}^{2}$, with x_obs_ and x_mod_, respectively observed and modelled plastic age distribution by decades (i) from the 1950s (*i* = 1) to 2000s (*i* = *n*).

**Supplementary Table 1: Plastic production market share per sector and associated mean and standard deviation used to compute the normal distribution for lifetime of different plastic objects.** Using global plastic production figures with market sector shares and lifetimes as presented in [1], we compute the discarded plastic population age distribution every year from 1950 to 2015.

| **Market sector** | **Market Share (%)** | **Mean lifetime**  **(years)** | **Standard deviation lifetime (years)** |
| --- | --- | --- | --- |
| Packaging | 31.2 | 1 | 0.4 |
| Transportation | 5.1 | 13 | 3 |
| Building & Construction | 8.0 | 35 | 7 |
| Electrical & Electronic | 2.0 | 8 | 2 |
| Consumer & Institutional Products | 9.4 | 3 | 1 |
| Industrial Machinery | 0.8 | 20 | 3 |
| Other | 9.3 | 5 | 1.5 |

**REFERENCES**

| [1] | Geyer, R., Jambeck, J. R. & Law, K. L. Production, use, and fate of all plastics ever made. *Science Advances* **3**, e1700782 (2017). |
| --- | --- |
